# A machine learning model for automated anesthesia risk classification in lumbar spinal stenosis patients: development and multicenter validation

**DOI:** 10.3389/fmed.2026.1910920

**Published:** 2026-08-26

**Authors:** Jitao Yang, Yixi Wang, Qihao Chen, Ning Ma, Paerhati Rexiti

**Affiliations:** 1 Department of Minimally Invasive Spine Surgery and Precision Orthopedics, The First Affiliated Hospital of Xinjiang Medical University, Urumqi, China; 2 Department of Anesthesiology, The First Affiliated Hospital of Xinjiang Medical University, Urumqi, China

**Keywords:** anesthetic risk, ASA classification, lumbar spinal stenosis, machine learning, multicenter study

## Abstract

**Background:**

Patients with lumbar spinal stenosis are typically elderly with multiple comorbidities, necessitating accurate preoperative anesthetic risk assessment. The American Society of Anesthesiologists (ASA) classification quantifies functional reserve and disease burden, serving as a widely used tool for risk stratification. However, ASA classification is often influenced by subjective factors including physician experience and varies among clinicians with different seniority, while the assessment process remains time-consuming. This study aimed to develop an automated model for anesthetic risk stratification and evaluate its performance, with the goal of providing decision support for surgical and anesthetic management in this patient population.

**Method:**

Clinical data of 600 patients with lumbar spinal stenosis were collected and randomly divided into training (*n* = 480) and internal validation (*n* = 120) sets. An additional 100 patients from another tertiary hospital formed an external validation set. The model was validated and hyperparameter-tuned using k-fold cross-validation. Model performance, including overall classification and high-risk identification, was evaluated using accuracy, macro-average precision, macro-average recall, macro-average F1 score, weighted Kappa, linear weighted accuracy, positive predictive value, and negative predictive value.

**Results:**

In the internal validation set, the accuracy, macro-average precision, macro-average recall, macro-average F1 score, weighted Kappa coefficient and linear weighted accuracy of the model are 0.97, 0.96, 0.95, 0.96, 0.93 and 0.98 respectively, while in the external validation set, they are 0.97, 0.97, 0.94, 0.96, 0.93 and 0.99 respectively. The confusion matrix heatmap shows that the error is mainly concentrated between adjacent classes, and there is no cross-class misjudgment. In the internal validation set, the model's accuracy, sensitivity, specificity, positive predictive value, negative predictive value, and Kappa coefficient for identifying high-risk patients were 0.98, 0.95, 0.98, 0.91, 0.99, and 0.92, respectively. In the external validation set, these values were 0.98, 0.93, 0.99, 0.93, 0.99, and 0.92, respectively.

**Conclusion:**

The machine learning model developed in this study demonstrates strong capability in stratifying anesthetic risk for patients with lumbar spinal stenosis, providing valuable reference for selecting surgical and anesthetic approaches.

## Introduction

Lumbar spinal stenosis (LSS) is the second leading cause of low back and leg pain in middle-aged and elderly individuals. It predominantly affects the elderly population, with an incidence rate of 5.1% to 10.0% (1–3). When conservative treatments prove ineffective or yield poor outcomes, surgical intervention may be considered (4, 5). Different surgical procedures require different types of anesthesia. For example, spinal fusion must be performed under general anesthesia, while decompression alone can be performed under local anesthesia. These patients are typically elderly and have multiple underlying medical conditions, with a wide range of comorbidities affecting the cardiovascular, pulmonary, renal, and metabolic systems, among others. Their physiological reserve and compensatory capacity in response to stress are significantly diminished. This objective reality makes their anesthetic management highly challenging, requiring that preoperative risk assessments accurately reflect their overall health status rather than focusing solely on localized spinal pathology.

The American Society of Anesthesiologists (ASA) classification is one of the most widely used tools for assessing overall preoperative health in the medical field today (6). By systematically integrating the severity of patients' comorbidities and their impact on daily functioning, this classification system provides a comprehensive reflection of patients' overall physiological reserve and translates the burden of complex multisystem diseases into an ordered risk hierarchy, thereby enabling an accurate assessment of preoperative status. The results of this classification are frequently used as a key reference for decision-making regarding the timing of surgery, the choice of anesthesia, and perioperative management strategies (7, 8).

In actual clinical practice, the determination of ASA classification relies heavily on the evaluating physician's comprehensive assessment of the patient's overall condition. A physician's personal experience, cognitive biases, and professional training background often result in significant variability in classification outcomes. When treating the same patient, physicians with different levels of experience often have clear differences in their assessment of key factors such as the severity of comorbidities, functional reserve, and compensatory capacity. Junior physicians, in particular, are prone to systematically overestimating or underestimating these factors.

In recent years, artificial intelligence (AI) technology (9, 10), particularly machine learning, has played a crucial role in medical diagnosis, prognosis assessment (11, 12), and other areas. Meng et al. developed a convolutional neural network measurement system using anteroposterior and lateral x-ray images from 1,682 patients with scoliosis. The results showed that the system achieved an accuracy rate of 89.7%–93.7%, and the measurement time was significantly shorter than that of manual measurement. Ying et al. (13) performed a retrospective study and developed a machine learning model capable of predicting various postoperative infections in elderly patients after cervical spine surgery, thereby offering a multiclass risk stratification tool to aid clinical decision-making. Using clinical data from 295 patients with spinal deformities, Balaban et al. (14) developed a predictive model for postoperative mechanical complications. The model achieved an accuracy rate of 72% and can provide effective support for preoperative risk stratification and surgical decision-making. However, no studies have yet explored the application of machine learning in assessing anesthesia risks for patients with LSS.

This study conducted a retrospective analysis of clinical data from 700 patients with LSS admitted to two tertiary hospitals, integrating multidimensional preoperative information including demographic characteristics, underlying medical conditions, laboratory test results, and functional status. Using machine learning algorithms, we constructed an automated anesthesia risk stratification model and evaluated its performance. The aim is to address the current challenges in clinical practice—namely, the high subjectivity, low inter-rater agreement, and cumbersome process associated with manual ASA classification, and to provide an objective reference for selecting anesthesia methods and surgical plans for these high-risk patients, who are often elderly and have multiple comorbidities.

## Materials and methods

### General information

This study followed the Declaration of Helsinki and was approved by our hospital. During the collection of raw data, patients' identifying information was omitted, and the ethics committee approved a waiver of informed consent for the study (permit numbers: 20260306-84).

Inclusion Criteria: (1) Age ≥ 18 years; (2) Confirmed diagnosis of LSS based on clinical presentation, physical examination, and imaging studies; (3) Underwent surgical treatment after failure of standard conservative therapy, with complete clinical records.

Exclusion Criteria: (1) History of prior spinal or joint surgery; (2) Concurrent spinal infection, tumor, fracture, or other conditions; (3) Presence of absolute contraindications for surgery; (4) Incomplete medical records.

Based on the inclusion and exclusion criteria outlined above, clinical data were collected from 600 patients admitted to our hospital between October 2024 and December 2025. These cases were randomly divided into a training set (480 cases) and an internal validation set (120 cases) at an 8:2 ratio.

### Anesthesia risk assessment

Two anesthesiologists independently performed preoperative ASA classification for patients with LSS, with neither anesthesiologist aware of the other's assessment results. For cases with inconsistent grading, a senior anesthesiologist will serve as an arbitrator, and the final class will be determined after all anesthesiologists reach a consensus through discussion. Prior to arbitration, the weighted Kappa coefficient was used to assess the agreement between the two anesthesiologists.

Specific classification criteria are as follows: Class I: Healthy patients with good tolerance to anesthesia and surgery; Class II: Patients with minor systemic diseases without substantial organ function limitations, capable of tolerating general anesthesia and surgery with minimal risk; examples include patients who smoke or drink alcohol, or those with well-controlled diabetes or hypertension.; Class III: Patients with high systemic disease and substantial organ function limitations, presenting one or more moderate to high conditions, where surgery and anesthesia carry significant risk, e.g., 3 months after the implantation of a pacemaker or a coronary stent; Class IV: Patients with severe systemic disease posing a threat to life, e.g., myocardial infarction or cerebrovascular accident within the past 3 months, severe decline in cardiac ejection fraction; Class V: Terminally ill patients with no prospect of survival without surgery; Class VI: Patients declared brain dead, prepared for organ procurement and transplantation.

### Included clinical characteristics

The clinical characteristics included in this study were determined based on the ASA physical status classification system and encompassed baseline demographic characteristics, lifestyle factors, and the functional status of major organ systems, as detailed below:

(1) Baseline Characteristics and Lifestyle Habits: Patient age, body mass index (BMI), prior surgical history, smoking history, and alcohol consumption history were recorded. Alcohol consumption history was categorized as light drinking, alcohol dependence, or heavy drinking.
(2) Cardiovascular System: A history of hypertension and blood pressure control were recorded. A history of coronary artery disease, myocardial infarction, cerebrovascular accident, transient ischemic attack, pacemaker implantation, and coronary stent implantation was documented. The time interval from the onset of these events to the time of assessment was specified and stratified evaluation was performed using a cutoff of three months. Cardiac valve function and ejection fraction were recorded.
(3) Respiratory System: History of pulmonary diseases, including mild pulmonary diseases, chronic obstructive pulmonary disease, and acute respiratory distress syndrome, etc., was recorded.
(4) Metabolic and Endocrine Systems: History of diabetes and blood glucose control levels were recorded. Lipid profile was also documented.
(5) Digestive System: Determine whether active hepatitis is present and record history of alcohol dependence or alcohol abuse.
(6) Urinary System: Assess kidney function and determine whether the patient is undergoing regular dialysis treatment.
(7) Special Physiological Conditions: Document whether the patient is pregnant.

### Design of the external validation set

The clinical data of 100 LSS patients admitted to another tertiary hospital in the same period were collected as the external validation set and the same inclusion and exclusion criteria were adopted. All ASA classifications were independently evaluated by the same two anesthesiologists with a unified scheme, and the evaluators were blind to each other.

### Construction of an automated anesthesia risk grading model

**Figure 1:**
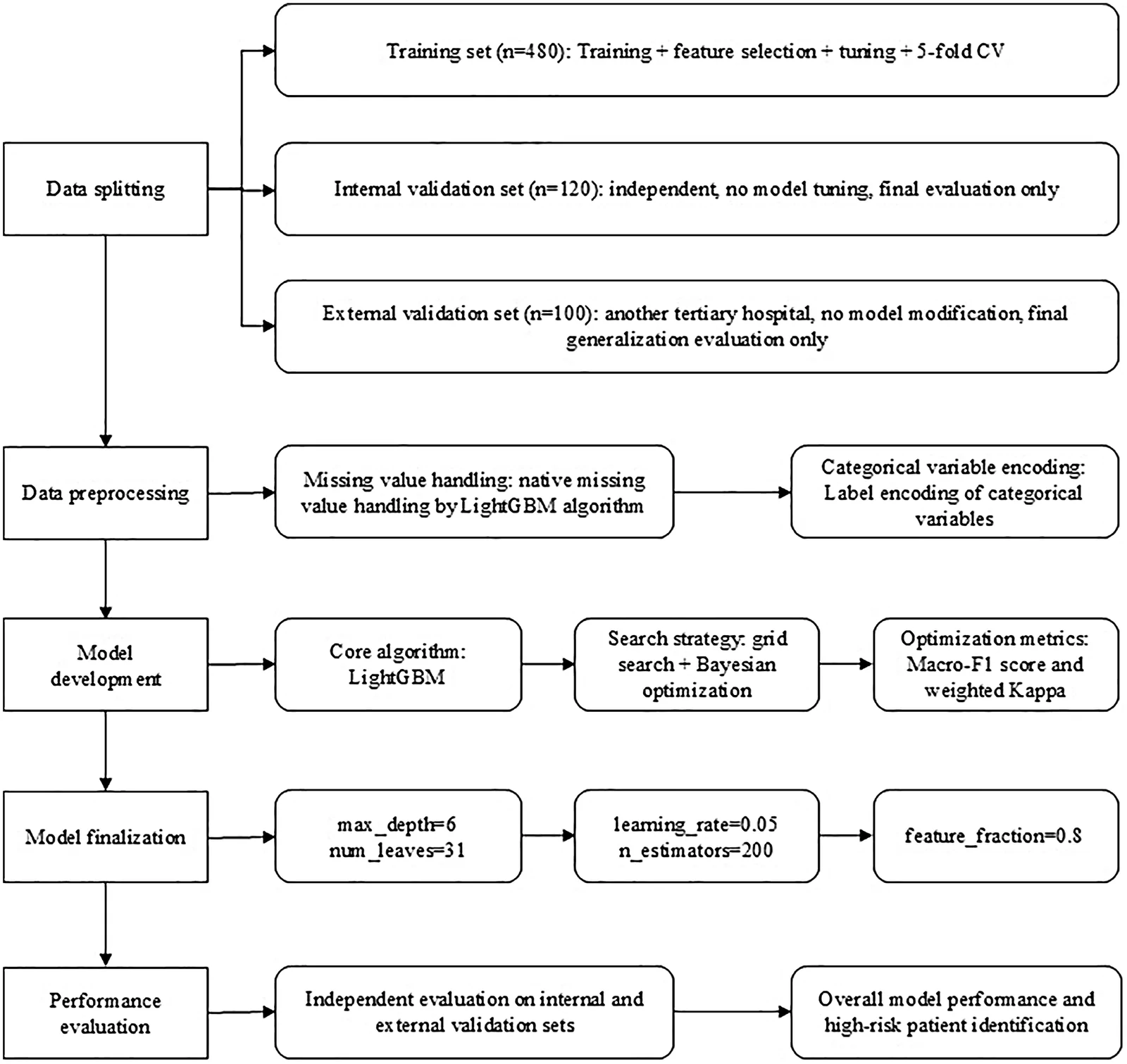
Workflow diagram of the LightGBM-based model development and validation process. A total of 700 patients were divided into training (*n* = 480), internal validation (*n* = 120), and external validation (*n* = 100) sets. The training set was used for feature selection, 5-fold cross-validation, and hyperparameter tuning (grid search + Bayesian optimization) with LightGBM. The internal and external validation sets were used exclusively for final performance evaluation and generalization assessment.

(1) Data Partitioning and Data Leakage Prevention Mechanisms: The multicenter clinical data included in this study were strictly partitioned into a training set (used for model training and hyperparameter optimization), an internal validation set, and an external validation set. The train_test_split function was used with the stratify parameter set to the ASA classification labels, ensuring that the proportions of ASA I, II, and III cases remained consistent across the training and internal validation sets. The external validation set comprised consecutive patients from another tertiary hospital. To ensure the objectivity and unbiasedness of the evaluation results, a uniform random seed (random_state=42) was fixed for all stages involving random data partitioning. At the same time, a rigorous data processing pipeline was established: all preprocessing parameters (such as encoding mappings for categorical variables and criteria for handling missing values) were calculated and extracted independently based solely on the training set, and then applied impartially to both the internal and external validation sets using the same standards, thereby completely eliminating any form of data leakage from the process. The internal and external validation sets remain completely “isolated” during the model training, hyperparameter tuning, and feature selection phases, and are never involved in any model selection process.
(2) Missing Value Handling and Feature Encoding: This study fully leveraged the LightGBM framework's native, efficient mechanism for handling missing values (Algorithm-native missing value handling). During training and prediction, the algorithm automatically identified the optimal branching paths without performing any manual or subjective interpolation on the original clinical data with missing values, thereby maximizing the integrity and objectivity of the real-world clinical data. For categorical variables in the baseline features (such as smoking history and history of systemic diseases), integer label encoding was uniformly applied before inputting them into the model.
(3) Handling Class Imbalance: Given the objective reality of an imbalanced distribution of ASA classification samples in actual clinical practice, this study introduced an adaptive class weight adjustment strategy into the LightGBM loss function (by setting class_weight = balanced to dynamically optimize scale_pos_weight). During model training, this mechanism assigns higher penalty weights to samples from the minority class, thereby effectively preventing the model from favoring the majority class.
(4) Cross-Validation and Hyperparameter Optimization Strategy: Hyperparameter optimization and internal validation were strictly conducted within the training set using 5-fold cross-validation. Using the Python-based LightGBM package (v4.x), a strategy combining grid search and Bayesian optimization was employed to perform global optimization of the hyperparameter space. The Macro-F1 Score and weighted Kappa coefficient were used as the core optimization metrics to guide hyperparameter selection, and an early stopping mechanism (early_stopping_rounds = 50) was introduced to effectively prevent model overfitting. Following cross-validation-based optimization, the final core hyperparameters determined for the model are: maximum tree depth = 6, number of leaf nodes = 31, learning rate = 0.05, number of decision trees = 200, and feature sampling rate = 0.8 (Figure 1).

### Model deployment and web tool

To facilitate clinical application, the final LightGBM model was deployed as a freely accessible web-based tool. The tool requires users to input the clinical variables described above and returns the predicted ASA classification in real time. The web tool is available at: https://asa.xjzyxd.cn/.

### Observation metrics

In the validation set, the overall model performance was evaluated using accuracy, macro-average precision, macro-average recall, and macro-average F1 score. The consistency between the model and expert-assigned ASA classification was assessed using weighted Kappa coefficient and linear weighted accuracy. Additionally, and confusion matrix heatmaps were plotted. The model's ability to identify high-risk patients was evaluated using accuracy, sensitivity, specificity, positive predictive value, negative predictive value, and the Kappa coefficient.

### Statistical analysis

Data normality was assessed using the Shapiro–Wilk test. Normally distributed quantitative data are presented as mean ± standard deviation ($\bar{x}\pm s$). Intergroup comparisons were performed using one-way analysis of variance (ANOVA). Categorical data are expressed as rates (%). The chi-square test was applied, with *p* < 0.05 indicating statistically significant differences. 95% confidence intervals (95% CI) of all performance indicators are calculated by bootstrap resampling with 1,000 iterations.

To interpret the model's predictions, we performed Shapley Additive Explanations (SHAP) analysis using the Treeexplainer. Feature importance was ranked by the mean absolute SHAP value across all samples, and features were subsequently grouped into clinical categories for system-level interpretation.

## Results

### General characteristics among all patients

There were no significant differences between the training set and the internal validation set or external validation set in terms of gender, age, BMI, prior surgery history, number of comorbidities, or preoperative ASA classification (*p* > 0.05) (Table 1).

**Table 1:** General characteristics among all patients.

Characteristic	Training set	Internal validation set	External validation set	Statistic	*p*-value
Gender					
Male	233 (48.5%)	59 (49.2%)	43 (43.0%)	*x^2^* *=* 1.118	0.57
Female	247 (51.5%)	61 (50.8%)	57 (57.0%)		
Age (years)	53.79 ± 14.518	56.72 ± 13.922	54.51 ± 13.448	F = 2.024	0.13
BMI(kg/m^2^)	26.2 ± 3.9	25.6 ± 6.1	26.0 ± 4.1	F = 0.7	0.49
Prior surgical history					
Present	213 (44.4%)	50 (41.7%)	40 (40.0%)	*x^2^* *=* 0.61	0.74
Absent	267 (55.6%)	70 (58.3%)	60 (60.0%)		
Number of comorbidities					
0	56 (11.7%)	24 (20.0%)	7 (7.0%)	*x^2^* *=* 11.06	0.09
1	122 (25.4%)	24 (20.0%)	27 (27.0%)		
2	281 (58.5%)	64 (53.3%)	59 (59.0%)		
≥3	21 (4.4%)	8 (6.7%)	7 (7.0%)		
Preoperative ASA classification					
Class I	43 (9.0%)	12 (10.0%)	11 (11.0%)	*x^2^* *=* 1.28	0.87
Class II	341 (71.0%)	86 (71.7%)	74 (74.0%)		
Class III	96 (20.0%)	22 (18.3%)	15 (15.0%)		

### Consistency Among evaluators

In the internal validation set, the weighted Kappa coefficient for the two anesthesiologists was 0.93 (95% CI: 0.84–0.98), and in the external validation set, it was 0.91 (95% CI: 0.80–0.98).

### Model robustness and generalization

**Figure 2:**
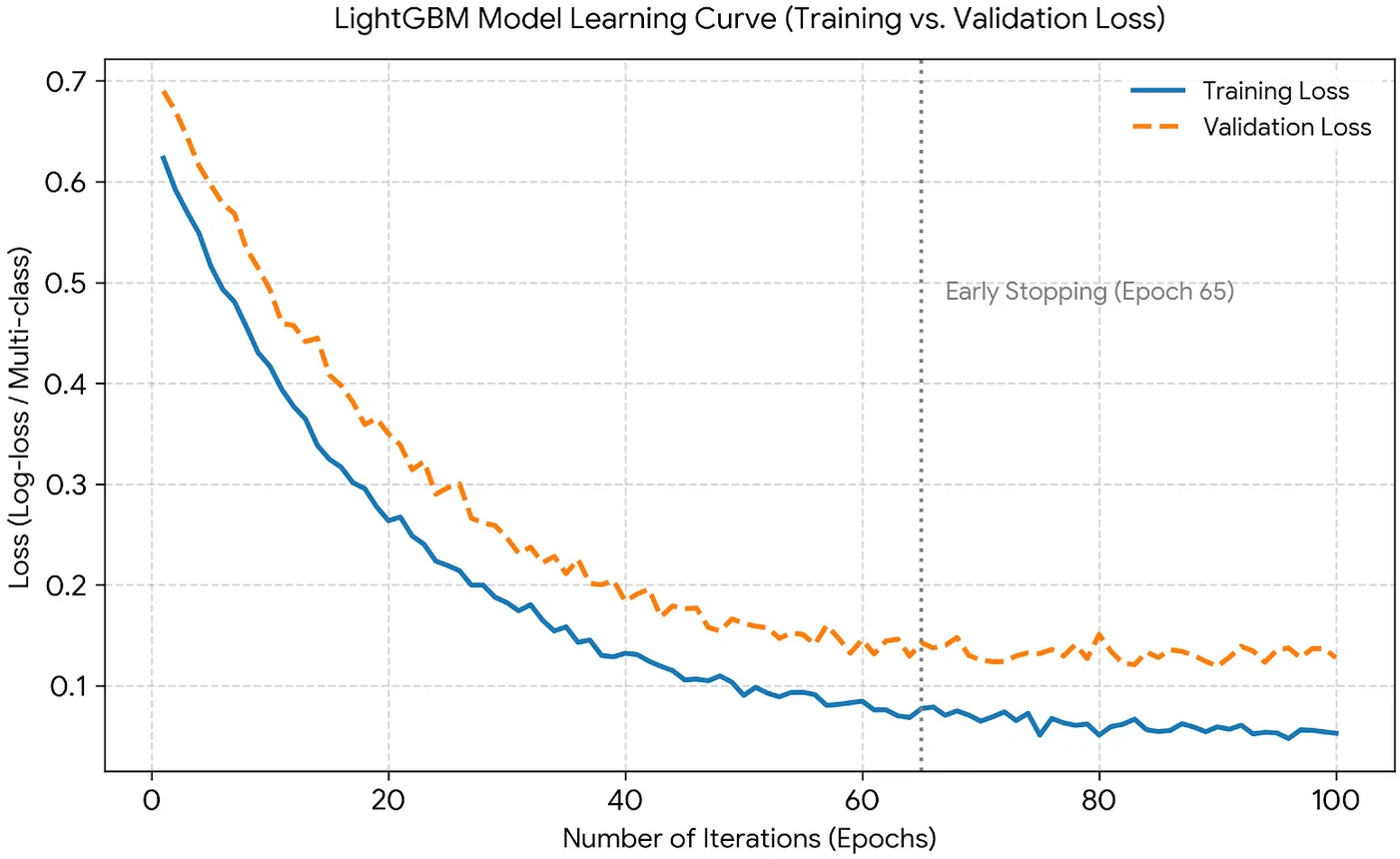
Learning curves of the LightGBM model. The learning curve shows that both the training loss (solid line) and the validation loss (dashed line) decrease steadily as the number of iterations increases, and then level off after convergence. The figure marks the early stopping points used in cross-validation (such as around the 65th iteration), at which the validation set loss reaches its minimum without overfitting, effectively ensuring the model's generalization ability.

In the 5-fold cross-validation phase, the model achieved a mean accuracy of 0.96 (range: 0.95–0.98). The learning curve shows that both the training loss and the validation loss decrease steadily as the number of iterations increases, and then level off after convergence. In about 65th iteration, the validation set loss reaches its minimum without overfitting (Figure 2).

### Overall model performance

**Figure 3:**
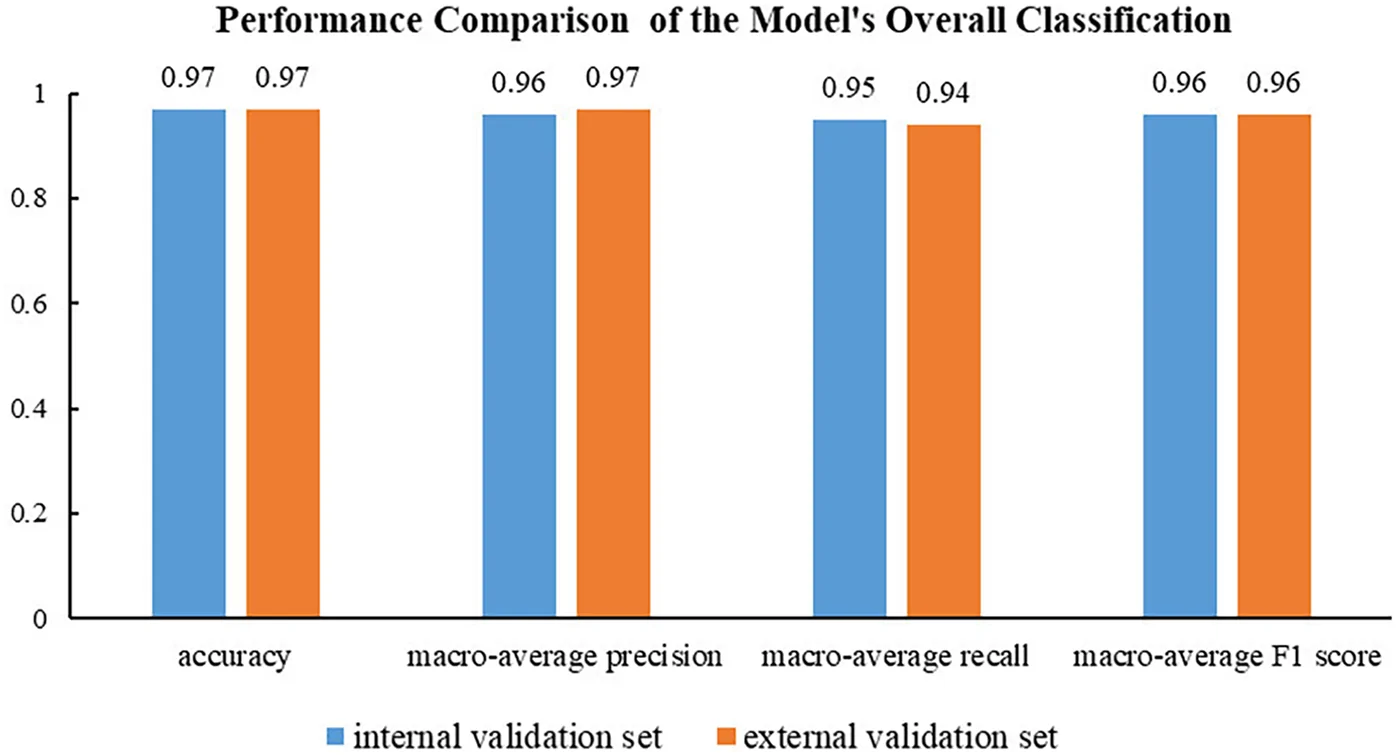
Overall classification performance of the machine learning model on internal and external validation sets. The performance metrics include accuracy, macro-average precision, macro-average recall, and macro-average F1 score. On the internal validation set, the accuracy, macro-average precision, macro-average recall, and macro-average F1 score were 0.97, 0.96, 0.95, and 0.96, respectively. On the external validation set, the values were 0.97, 0.97, 0.94, and 0.96, respectively. The highly consistent results between the two independent datasets demonstrate the model's robust generalizability and stable performance in grading anesthesia risk.

Using expert-assigned ASA classification results as a reference, a confusion matrix was constructed. On the internal validation set, the model achieved accuracy, macro-average precision, macro-average recall, and macro-average F1 score of 0.97 (95% CI: 0.93–0.99), 0.96 (95% CI: 0.92–1.00), 0.95 (95% CI: 0.87–1.00), and 0.96 (95% CI: 0.90–0.99), respectively. On the external validation set, these metrics were 0.97 (95% CI: 0.94–1.00), 0.97 (95% CI: 0.92–1.00), 0.94 (95% CI: 0.86–1.00), and 0.96 (95% CI: 0.89–1.00) (Figure 3) (Table 2).

**Table 2:** Overall performance of model.

Metric	Internal validation set	External validation set
Accuracy (95% CI)	0.97 (0.93–0.99)	0.97 (0.94–1.00)
Macro-average precision (95% CI)	0.96 (0.92–1.00)	0.97 (0.92–1.00)
Macro-average recall (95% CI)	0.95 (0.87–1.00)	0.94 (0.86–1.00)
Macro-average F1 score (95% CI)	0.96 (0.90–0.99)	0.96 (0.89–1.00)

### Consistency of model: weighted index based on ordered classification

Using expert-assigned ASA classification results as a reference, the model achieved a weighted Kappa coefficient of 0.93 (95% CI: 0.85–0.99) and a linearly weighted accuracy of 0.98 (95% CI: 0.97–1.00) in the internal validation set, while in the external validation set, these values were 0.93 (95% CI: 0.84–1.00) and 0.99 (95% CI: 0.97–1.00), respectively (Table 3).

**Table 3:** Consistency of model.

Metric	Internal validation set	External validation set
Weighted Kappa coefficient (95% CI)	0.93 (0.85–0.99)	0.93 (0.84–1.00)
Linearly weighted accuracy (95% CI)	0.98 (0.97–1.00)	0.99 (0.97–1.00)

**Figure 4:**
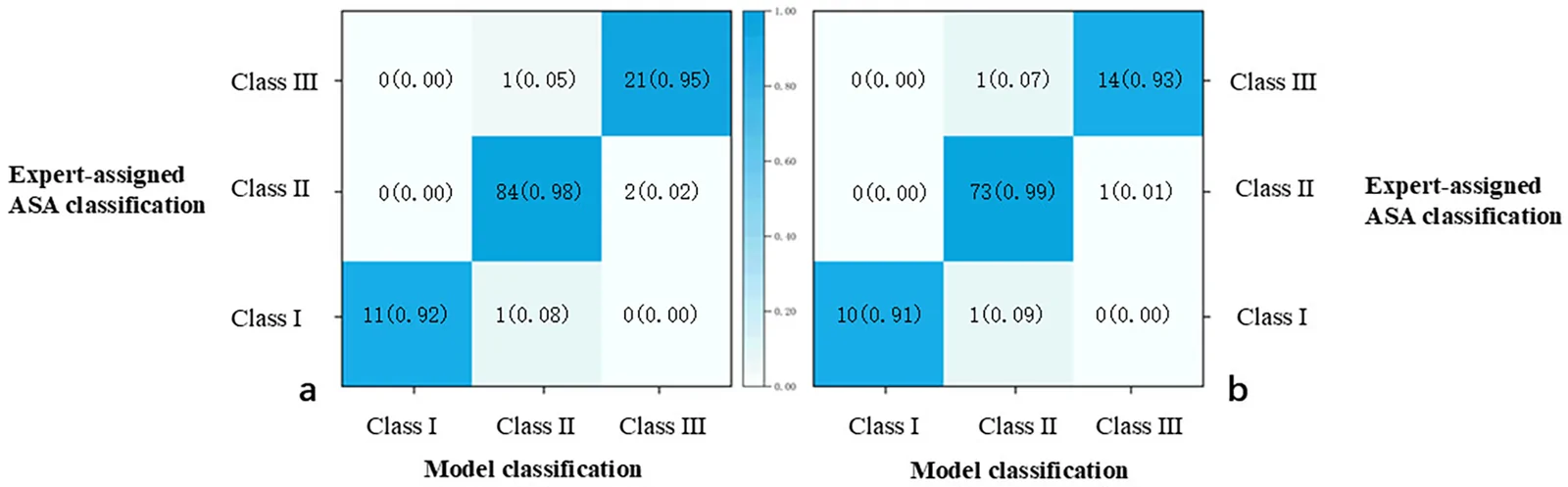
Confusion matrix heatmap of the machine learning model for ASA classification on the internal **(a)** and external validation set **(b)**. The rows represent the ground truth labels (ASA classification I, II, and III) assessed by anesthesiologists, and the columns represent the predicted labels by the machine learning model. Each cell displays the absolute case count and the corresponding row percentage (recall rate). The diagonal cells (shaded) indicate the proportions of correct classifications, demonstrating high overall accuracy. Notably, all misclassifications are confined to adjacent classes (e.g., Class Ⅰ misclassified as Class Ⅱ, Class Ⅲ misclassified as Class Ⅱ), with no cross-class errors (e.g., Class Ⅲ misclassified as Class Ⅰ). This pattern confirms the model's understanding of the ordinal nature of ASA classification and supports its clinical acceptability.

Plotting the confusion matrix heatmap reveals that errors in both the internal and external validation set predominantly cluster around adjacent classes, with no misclassifications across classes (Figure 4).

### Model's performance to identify high- risk

**Figure 5:**
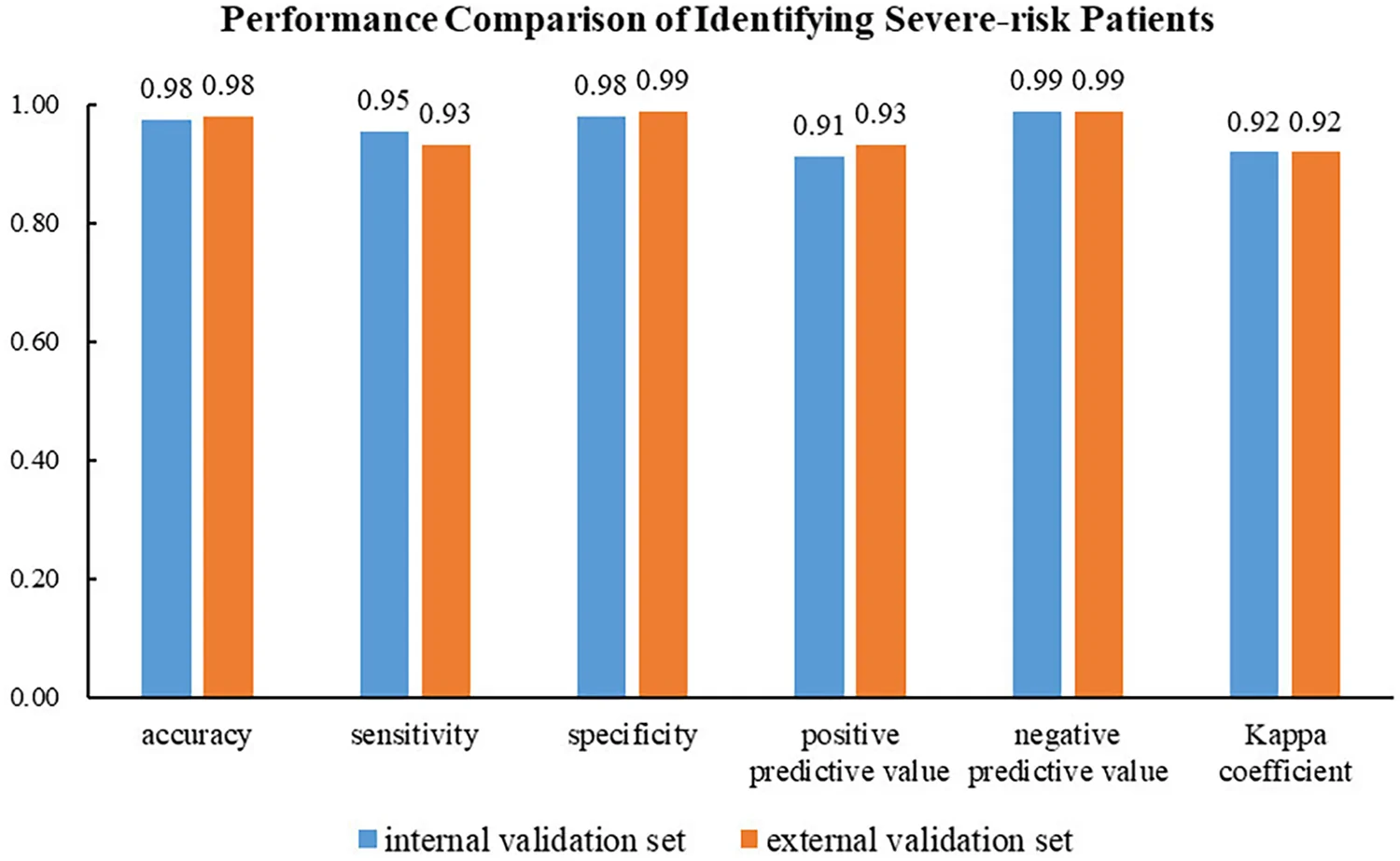
Performance of the machine learning model for identifying high-risk patients on internal and external validation sets. ASA classes I and II were defined as low risk, while Class III was defined as high-risk. The model's performance was evaluated using accuracy, sensitivity, specificity, positive predictive value (PPV), negative predictive value (NPV), and Kappa coefficient. On the internal validation set (*n* = 120), the model achieved an accuracy of 0.98, sensitivity of 0.95, specificity of 0.98, PPV of 0.91, NPV of 0.99, and Kappa of 0.92. On the external validation set (*n* = 100), the corresponding values were 0.98, 0.93, 0.99, 0.93, 0.99, and 0.92. The consistently high performance across both datasets indicates that the model has good generalizability in distinguishing high-risk from low-risk patients, with excellent negative predictive value (0.99) and substantial to almost perfect agreement (Kappa > 0.90).

ASA classes I and II were defined as low-risk, while Class III was defined as high- risk. In the internal validation set, the model achieved an accuracy of 0.98 (95% CI: 0.94–1.00), a sensitivity of 0.95 (95% CI: 0.85–1.00), a specificity of 0.98 (95% CI: 0.95–1.00), a positive predictive value of 0.91 (95% CI: 0.79–1.00), a negative predictive value of 0.99 (95% CI: 0.97–1.00), and a Kappa coefficient of 0.92 (95% CI: 0.82–1.00). In the external validation set, these metrics were 0.98 (95% CI: 0.95–1.00), 0.93 (95% CI: 0.77–1.00), 0.99 (95% CI: 0.96–1.00), 0.93 (95% CI: 0.78–1.00), 0.99 (95% CI: 0.96–1.00), and 0.92 (95% CI: 0.79–1.00), respectively (Figure 5) (Table 4).

**Table 4:** Model's ability to identify high-risk.

Metric	Internal validation set	External validation set
Accuracy (95% CI)	0.98 (0.94–1.00)	0.98 (0.95–1.00)
Sensitivity (95% CI)	0.95 (0.85–1.00)	0.93 (0.77–1.00)
Specificity (95% CI)	0.98 (0.95–1.00)	0.99 (0.96–1.00)
Positive predictive value (95% CI)	0.91 (0.79–1.00)	0.93 (0.78–1.00)
Negative predictive value (95% CI)	0.99 (0.97–1.00)	0.99 (0.96–1.00)
Kappa coefficient (95% CI)	0.92 (0.82–1.00)	0.92 (0.79–1.00)

### Feature importance analysis

**Figure 6:**
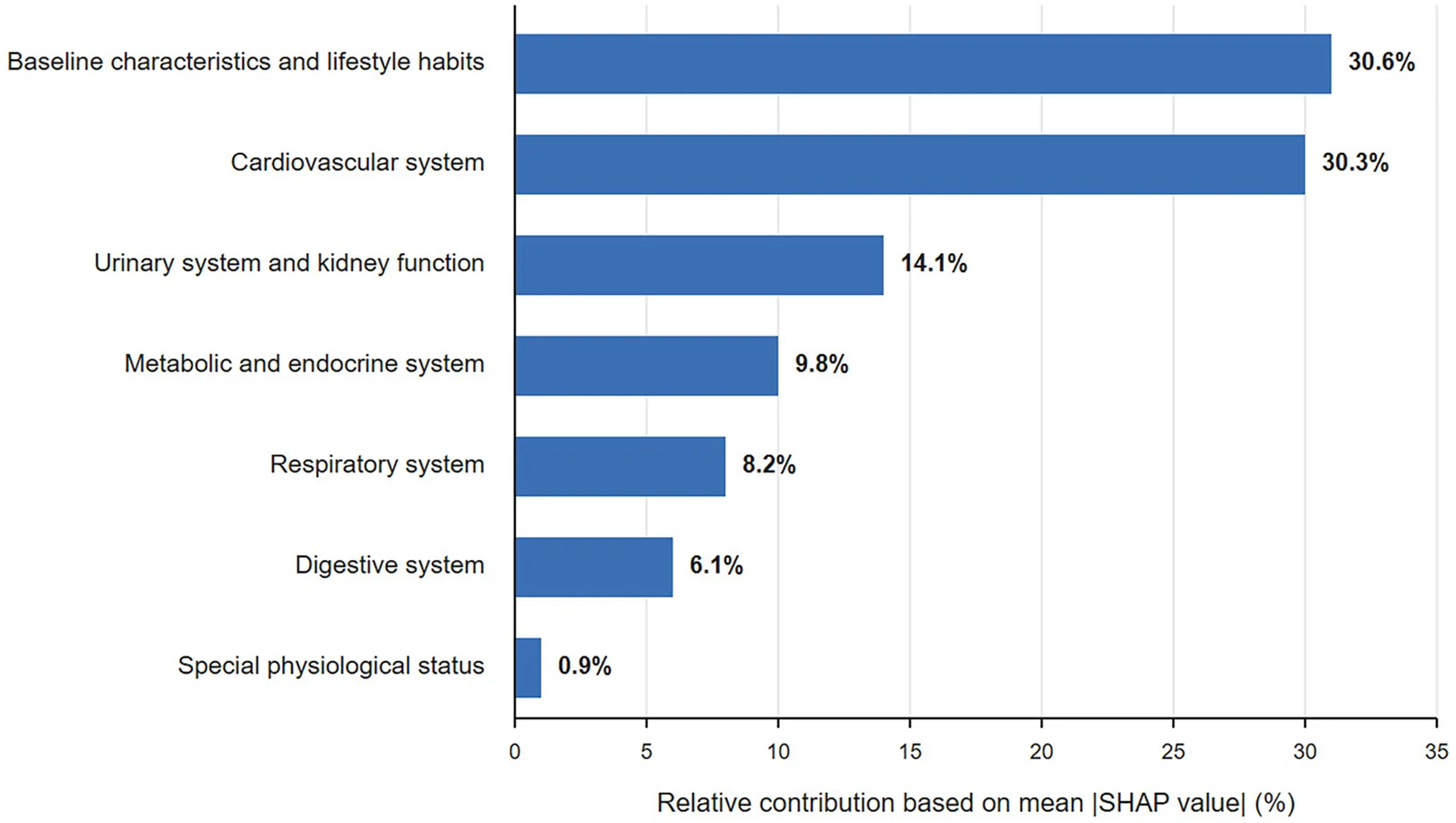
Relative contribution of clinical feature categories to ASA classification prediction based on SHAP analysis. Features were grouped into seven clinical categories according to their physiological systems. The contribution of each category was calculated as the mean absolute SHAP value across all samples, expressed as a percentage of the total. Baseline characteristics and lifestyle habits (30.6%) and cardiovascular system (30.3%) were the two dominant contributors, together accounting for over 60% of the model's predictions.

SHAP analysis grouped by clinical categories revealed that baseline characteristics and lifestyle habits (30.6%) and cardiovascular system (30.3%) were the two dominant contributors, together accounting for more than 60% of the model's predictions. Urinary system and kidney function (14.1%), metabolic and endocrine system (9.8%), respiratory system (8.2%), digestive system (6.1%), and special physiological status (0.9%) contributed to the remaining proportion (Figure 6).

## Discussion

Patients with LSS are characterized by advanced age, multiple underlying conditions, and high surgical risks, necessitating careful preoperative assessment of anesthetic risks (10, 15–18). Rapid and precise ASA classification aids clinicians in objectively evaluating patients' overall status, developing personalized anesthesia and surgical plan (19).

In recent years, AI technology, especially machine learning, has played a significant role in preoperative patient assessment, treatment recommendations, and complication prediction (20–23). Various models have been reported, including automated American Spinal Injury Association(ASIA) classification for spinal cord injury patients and intelligent diagnosis of cervical spine fractures. Using computed tomography data from 219 patients with cervical spine fractures, Van et al. (24) developed an AI-based diagnostic assistance model. This model significantly improves diagnostic accuracy by increasing the sensitivity of radiologists by 10.5% (to 98.6%) and specificity by 0.8% (to 100%), while keeping the additional cost at just 0.3%. Jillala et al. (25) developed a model to predict ASIA classification in patients with spinal cord injury using five machine learning algorithms (random forest, linear discriminant analysis, k-nearest neighbors, naive Bayes, and support vector machines). The results showed that the random forest algorithm performed best, achieving an accuracy rate of 81.7%. Lei et al. (26) developed and externally validated a machine learning-based risk prediction model for postoperative pulmonary complications in elderly patients undergoing degenerative spine surgery, which may facilitate perioperative risk stratification. Meier et al. (27) created a clinical decision support system that analyzes postoperative physiological data in real time and promptly provides medication adjustment recommendations.

To address the issues of high subjectivity, poor inter-rater agreement, and cumbersome, time-consuming processes associated with manual ASA classification, our team developed an automated anesthesia risk classification model using machine learning technology and clinical data from 700 patients across multiple centers. The model aims to streamline the risk assessment process, improve assessment accuracy, and provide a reference for the scientific and rational selection of anesthesia and surgical procedures.

LightGBM was selected as the core algorithm due to its efficiency, stability, and interpretability in handling structured clinical data (13, 28). It offers fast training speed and low memory usage, enabling real-time deployment on routine hospital servers. The algorithm also supports class-weight adjustment to address class imbalance, improving sensitivity for rare high-risk cases. Additionally, its built-in feature importance analysis facilitates clinicians' understanding of the model's decision logic and enhances trust. Based on its comprehensive advantages in performance, efficiency, interpretability, and clinical applicability, LightGBM was chosen as the primary model for this study.

The automated anesthesia risk classification model for LSS patients developed by our team was trained using clinical data from 480 patients and evaluated on a 120-case internal validation set and a 100-case external validation set. During the 5-fold cross-validation phase, the accuracy across folds ranged from 0.95 to 0.98, with a mean of 0.96, indicating stable performance. Subsequently, on the internal validation set (*n* = 120) and external validation set (*n* = 100), both of which were completely independent of the training data, the model achieved an accuracy of 0.97 in both cases, highly consistent with the cross-validation results. The learning curves further showed that both training loss and validation loss decreased steadily with increasing iterations and plateaued after convergence. Early stopping was applied at epoch 65 based on cross-validation performance, at which point the validation loss reached its optimum, with no divergence between training and validation losses. These findings indicate that the model generalizes well and does not suffer from overfitting.

Results of performance evaluation indicate that the model achieves an accuracy of 0.97, macro-average precision of 0.96, macro-average recall of 0.95, and a macro-average F1 score of 0.96. Similar outcomes were obtained in external validation set. These metrics demonstrate the model's high reliability, enabling the identification of as many patients as possible while ensuring the accuracy of risk assessment results.

Additionally, according to the precision results, when the model predicts a patient belongs to a specific ASA class, its accuracy rate reaches 96%–97%. In clinical practice, this means that when the model classifies a patient as “high-risk (ASA class III)”, the result is highly reliable. This enables clinicians to swiftly adopt its definitive high-risk alerts, initiate corresponding contingency plans, and enhance decision-making efficiency. Recall results indicate the model successfully identifies 94%–95% of patients genuinely belonging to each target class, demonstrating robust case capture capability. However, approximately 5%–6% of target patients remain missed or misclassified into other classes, representing a key area for future optimization.

A higher ASA classification indicates a higher anesthetic risk. Misclassifications across categories (e.g., misclassifying a Class III patient as Class I) pose a far greater safety risk than misclassifications between adjacent categories (e.g., misclassifying a Class III patient as Class II). Misclassification across risk categories implies a significant underestimation of a patient's actual anesthetic risk, which may lead to clinical hazards such as inadequate preanesthetic assessment, lack of emergency protocols, and inappropriate choice of surgical approach, thereby significantly increasing the risk of perioperative adverse events.

To assess the model's consistency with the ordered rankings, we calculated the weighted Kappa coefficient and the linear weighted accuracy. The results showed that the weighted Kappa coefficient reaches 0.93, while the linear weighted accuracy achieves 0.98–0.99. According to Landis & Koch's criterion, this indicates that the model achieves “almost perfect” with expert-assigned ASA classification (29, 30). The model does not perform random classification but highly adheres to the inherent hierarchical logic of the ASA classification system. The confusion matrix heatmap reveals that errors in both internal and external validation sets predominantly occur between adjacent severity levels. All prediction errors represent minor “one-grade-off” discrepancies (e.g., misclassifying Class II as Class I), effectively avoiding cross-class errors that could lead to severe clinical misjudgments. These findings indicate the model possesses good clinical acceptability. Even when isolated misclassifications occur, the error types are clinically lower-risk and more readily tolerable.

For assessing anesthesia risks, accurately identifying high-risk patients is of paramount importance (31–33). In this study, ASA Class I and II patients were defined as low-risk, while Class III patients were classified as high-risk. The results show that the model demonstrated excellent and consistent discriminatory performance in both the internal and external validation set. In the internal validation set, the model achieved an accuracy of 0.98, a sensitivity of 0.95, a specificity of 0.98, a positive predictive value of 0.91, a negative predictive value of 0.99, and a Kappa coefficient of 0.92. The results in the external validation set were similar. These results fully demonstrate that the model possesses good generalization ability and does not exhibit significant overfitting. When the model identifies a high-risk case, its accuracy exceeds 90%. Clinicians can rely on this assessment to further evaluate the patient's overall condition and carefully select the appropriate anesthesia and surgical plan. The Kappa coefficients were all 0.92, indicating “almost perfect agreement” between the model and the gold standard. This level of agreement was fully replicated across two independent datasets, further demonstrating the model's reliability.

Based on the above results, the authors believe that this model has clear potential as a clinical decision-making aid. However, the model still has a misdiagnosis rate of 5%–7%. In clinical practice, patients classified by the model as high-risk (Class III) can be considered a reliable risk warning and require close attention. For patients classified by the model as low-risk (Class I/II), especially those with complex comorbidities, undergoing major surgery, or presenting other clinical risk factors, clinicians must conduct an independent and comprehensive clinical assessment and avoid relying solely on the model.

It is worth noting that the consistency between this model and evaluations by specialist physicians (weighted Kappa: 0.93) is very close to the consistency between two anesthesiology specialists (weighted Kappa: 0.91–0.93). These results indicate that the model's consistency is approaching the level of inter-expert agreement, further confirming its outstanding performance in the ASA classification task. At the same time, this finding suggests that the model can serve as a standardized reference tool in clinical practice to reduce assessment variability among different physicians.

In this study, feature importance was evaluated using the built-in methods of the LightGBM model, based on both split gain and split frequency, to quantitatively rank all preoperative clinical features. SHAP analysis were further applied to interpret the direction and magnitude of each feature's contribution to the model's predictions. The SHAP analysis revealed that the core variables contributing most to ASA classification prediction, particularly for identifying high-risk patients, were patient age, cardiovascular comorbidities (especially coronary artery disease and its time interval to surgery), and kidney function status. This ranking of variable importance aligns closely with established clinical reasoning in anesthetic practice, confirming that the model relies on clinically relevant predictors rather than noise or confounding factors. Such transparent explanation of feature contributions enables clinicians to not only obtain an automated classification but also understand the primary drivers behind the prediction, thereby substantially enhancing the clinical acceptability and safety of the model.

This study aims to develop an automated anesthesia risk stratification model to serve as a clinical decision-support tool. This model is intended to provide guidance for the selection of surgical and anesthesia plans, while the final decision remains with the physician. In clinical practice, all input variables for the model consist of routine preoperative clinical data, covering demographic characteristics and medical history across various systems, without the need for additional tests or data entry. The model's predictions can be completed before anesthesia and surgical plans are finalized. Anesthesiologists and spinal surgeons can refer to the model output and incorporate it with the patient's overall clinical status to reach the final treatment decisions.

The authors believe that this model has high clinical value. First, the model exhibits high sensitivity for high-risk patients, effectively reducing the risk of missed diagnoses in this population and ensuring patient safety. Second, the model's outputs are standardized and highly reproducible, helping to minimize inconsistencies in assessments caused by variations in physician experience and providing an objective reference for less experienced physicians. Furthermore, since the model's input variables consist entirely of existing clinical data, no additional tests or data entry are required, thereby avoiding increased medical costs and clinical workload.

In summary, the automated anesthesia risk classification model for LSS patients proposed in this study demonstrated robust performance following multicenter validation. It can assist surgeons and anesthesiologists in making scientifically sound clinical decisions.

## Limitations

Several limitations of this study should be acknowledged. First, the retrospective design and the inclusion of patients from only two centers inevitably introduce selection bias. ASA class Ⅳ–Ⅵ patients were not included, so the model is only applicable to ASA class Ⅰ–Ⅲ patients undergoing elective surgery. Second, only a LightGBM model was developed, and no systematic comparison was made with other algorithms, leaving its incremental value unclear. Third, because the framework was designed to output discrete labels based on hard thresholds to facilitate direct decision making, conventional ROC and decision curve analyses are not fully applicable. Fourth, data on postoperative complications and other clinical outcomes were not collected, so the association between model-predicated ASA classifications and these hard endpoints needs further validation. Future multicenter prospective studies with larger cohorts and multi algorithm comparisons are warranted to confirm its clinical utility.

## Data Availability

The raw data supporting the conclusions of this article will be made available by the authors, without undue reservation.

## References

[1] Han G Zou D Li X Zhang S Li Z Zhou S. Can fat infiltration in the multifidus muscle be a predictor of postoperative symptoms and complications in patients undergoing lumbar fusion for degenerative lumbar spinal stenosis? A case-control study. J Orthop Surg Res. (2022) 17(1):289. 10.1186/s13018-022-03186-2 35619169 PMC9137055

[2] Kwon J- Moon S-H Park S-Y Park S-J Park S-R Suk K-S. Lumbar spinal stenosis: review update 2022. Asian Spine J. (2022) 16(5):789–98. 10.31616/asj.2022.0366 36266248 PMC9633250

[3] Wang F Wang R Zhang C Song E Li F. Clinical effects of arthroscopic-assisted uni-portal spinal surgery and unilateral bi-portal endoscopy on unilateral laminotomy for bilateral decompression in patients with lumbar spinal stenosis: a retrospective cohort study. J Orthop Surg Res. (2024) 19(1):167. 10.1186/s13018-024-04621-2 38444008 PMC10916320

[4] Tighe CA Bachrach RL Perera S Weiner DK. Insomnia symptoms and postoperative healthcare utilization in veterans undergoing decompressive laminectomy for lumbar spinal stenosis. Sleep Adv. (2023) 4(1):zpad005. 10.1093/sleepadvances/zpad005 37193289 PMC10108638

[5] Beresford-Cleary NJA Silman A Thakar C Gardner A Harding I Cooper C. Findings from a pilot randomized trial of spinal decompression alone or spinal decompression plus instrumented fusion. Bone Jt Open. (2023) 4(8):573–9. 10.1302/2633-1462.48.BJO-2023-0049 37549931 PMC10493898

[6] Ambati VS Patel A Dada A Macki M Chan AK Chou D. Do patients with high ASA grades benefit from CSM surgery?: a report from the quality outcomes database. Clin Spine Surg. (2025) 38(4):197–203. 10.1097/BSD.0000000000001774 40257936

[7] Langella F Barile F Bellosta-Lòpez P Fusini F Compagnone D Vanni D. Identifying key factors influencing hospital stay after spine surgery: a comprehensive predictive model. Glob. Spine J. (2025) 15(8):3756–63. 10.1177/21925682251331451 PMC11962937 40168554

[8] Quiceno E Seaman S Hussein A Dholaria N Pico A Abdulla E. Clinical outcomes and complication profile of spine surgery in septuagenarians and octogenarians: case series. World Neurosurg. (2024) 185:e878–85. 10.1016/j.wneu.2024.02.146 38453010

[9] Khalsa A, Peregoff J, Turlip RW, Zemberi J, Capone G, Kwon, M AI-Assisted, literature-informed development and retrospective validation of a point-based surgical site infection risk calculator for spine surgery. Glob. Spine J. (2026)16(6):2749–57. 10.1177/21925682251415176 PMC12774816 41490806

[10] Maity A Brown EDL McCann RA Karkare A Orsino EA Ghanekar SD. Advent of artificial intelligence in spine research: an updated perspective. J Clin Med. (2026) 15(2):820. 10.3390/jcm15020820 41598756 PMC12841851

[11] Almulihi Q Alquraini A Almulihi F Alzahid A Qahtani S Almulhim M. Applications of artificial intelligence and machine learning in emergency medicine triage—a systematic review. Med Arch. (2024) 78(3):198–206. 10.5455/msm.2024.236-239 39944197 PMC11813208

[12] Han SS Azad TD Suarez PA Ratliff JK. A machine learning approach for predictive models of adverse events following spine surgery. Spine J. (2019) 19(11):1772–81. 10.1016/j.spinee.2019.06.018 31229662

[13] Ying Y Fan Z Fu L He C Huang Z Teng H. Multiclass classification of infections after cervical spine surgery in the elderly: a machine learning approach based on preoperative and perioperative data. Eur Spine J. (2026) 35(3):1377–88. 10.1007/s00586-026-09761-z 41619022

[14] Balaban B Demirci N Yilgor C Yucekul A Zulemyan T Haddad S. Predicting mechanical complications in adult spinal deformity patients with postoperative proportioned and moderately disproportioned alignment. Acta Orthop Traumatol Turc. (2025) 59(4):210–21. 10.5152/j.aott.2025.24146 40728025 PMC12362533

[15] Rhee W Chang SY Chang B Kim H. Prediction of early postoperative complications and transfusion risk after lumbar spinal stenosis surgery in geriatric patients: machine learning approach based on comprehensive geriatric assessment. BMC Med Inform Decis Mak. (2025) 25(1):279. 10.1186/s12911-025-03125-1 40722012 PMC12306017

[16] Lin W Liu J Zhan Z. Study on influencing factors of postoperative complications in elderly patients with lumbar spinal stenosis. Medicine (Baltimore). (2025) 104(8):e41476. 10.1097/MD.0000000000041476 39993119 PMC11856973

[17] Alhaug OK Dolatowski FC Lønne G. Response to letter to the editor regarding “predictors for failure after surgery for lumbar spinal stenosis: a prospective observational study”. Spine J. (2023) 23(12):1944. 10.1016/j.spinee.2023.08.022 38007252

[18] Shui M Zhao D Xue Z Wu A. Impact of spinal/epidural anesthesia versus general anesthesia on perioperative outcomes in patients undergoing lumbar spine surgery: an updated systematic review and meta-analysis. Clin Spine Surg. (2023) 36(6):227–36. 10.1097/BSD.0000000000001374 35943881

[19] Schupper AJ Shuman WH Baron RB Neifert SN Chapman EK Gilligan J. Utilization of the American society of anesthesiologists (ASA) classification system in evaluating outcomes and costs following deformity spine procedures. Spine Deform. (2021) 9(1):185–90. 10.1007/s43390-020-00176-4 32780301

[20] Yasheng P Yusufu A Yimiti Y Luan H Peng C Song X. Web-based machine learning application for interpretable prediction of prolonged length of stay after lumbar spinal stenosis surgery: a retrospective cohort study with explainable AI. Front Physiol. (2025) 16:1542240. 10.3389/fphys.2025.1542240 40046179 PMC11880216

[21] Mehmet S Elmarawany MN Harding I Bowey AJ Andrews J Chan D. AI Versus the spinal surgeons in the management of controversial spinal surgery scenarios. Eur Spine J. (2025) 34(9):3736–46. 10.1007/s00586-025-08825-w 40175645

[22] Mani K Scharfenberger T Goldman SN Kleinbart E Mostafa E De La Garza Ramos R. Multimodal machine learning for predicting perioperative safety indicators in spinal surgery. Spine J. (2025) 25(11):2450–60. 10.1016/j.spinee.2025.03.021 40164437

[23] Han H Li R Fu D Zhou H Zhan Z Wu Y’. Revolutionizing spinal interventions: a systematic review of artificial intelligence technology applications in contemporary surgery. BMC Surg. (2024) 24(1):345. 10.1186/s12893-024-02646-2 39501233 PMC11536876

[24] Van Den Wittenboer GJ Nijholt IM Kvamme I Van Lieshout C Maas M Boomsma MF. Potential change in healthcare costs of implementing artificial intelligence for detecting cervical spine fractures on CT: an early health technology assessment. Eur Radiol. (2026) 36(6):5182–90. 10.1007/s00330-025-12255-z 41489842

[25] Jillala RR Aude CA Vattipally VN Ran KR Jiang K Weber-Levine C. Assessing and validating machine learning-enhanced imputation of admission American spinal injury association impairment scale grades for spinal cord injury. J Neurosurg Spine. (2025) 43(1):90–7. 10.3171/2025.1.SPINE241135 40344756

[26] Lei R Ou J Chen Y Luo M Ruan X Wei J. Development and external validation of an explainable machine-learning model for predicting postoperative pulmonary complications in older adults undergoing degenerative spine surgery. J Clin Med. (2026) 15(14):5375. 10.3390/jcm15145375 42513290 PMC13412764

[27] Meier TA Refahi MS Hearne G Restifo DS Munoz-Acuna R Rosen GL. The role and applications of artificial intelligence in the treatment of chronic pain. Curr Pain Headache Rep. (2024) 28(8):769–84. 10.1007/s11916-024-01264-0 38822995

[28] Dor O Haim O Azolai L Agur A Cohen M Shomron N. Automatic detection of spinal pathologies based on MRI scans. Eur Spine J. (2026) 35(3):1345–53. 10.1007/s00586-025-09547-9 41258976

[29] Sadiqi S De Gendt EEA Muijs SPJ Post MWM Benneker LM Holas M. Validation of the AO spine CROST (clinician reported outcome spine trauma) in the clinical setting. Eur Spine J. (2024) 33(4):1607–16. 10.1007/s00586-024-08145-5 38367026

[30] Zhang M Li J Zhou Y Chen Z Wang P Hu B. Generative AI in degenerative lumbar spinal stenosis care: a NASS guideline-compliant comparative analysis of ChatGPT and DeepSeek. J Orthop Surg (Hong Kong). (2025) 33(3):10225536251407382. 10.1177/10225536251407382 41353581

[31] Patil H Garg N Navakar D Banabokade L. Lumbar spine surgeries under spinal anesthesia in high-risk patients: a retrospective analysis. World Neurosurg. (2019) 124:e779–82. 10.1016/j.wneu.2019.01.023 30682512

[32] Ahsan T Wang AY Karimi H Kanter MJ Olmos M Kosarchuk JJ. Assessing the safety and efficacy of spinal anesthesia in patients with significant comorbidities. World Neurosurg. (2023) 177:e110–7. 10.1016/j.wneu.2023.05.116 37295471

[33] Garg B Ahuja K Sharan AD. Regional anesthesia for spine surgery. J Am Acad Orthop Surg. (2022) 30(17):809–19. 10.5435/JAAOS-D-22-00101 35617645

